# Displaying wastewater surveillance data: an ethics framework

**DOI:** 10.1093/jlb/lsaf001

**Published:** 2025-06-09

**Authors:** Govind Persad, Anne Barnhill, Douglas MacKay

**Affiliations:** Sturm College of Law, University of Denver, Denver, CO, USA; Berman Institute of Bioethics, Johns Hopkins University, Baltimore, MD, USA; Public Policy, The University of North Carolina at Chapel Hill, Chapel Hill, NC, USA

The COVID-19 pandemic prompted renewed interest in studying wastewater to identify the presence of pathogens. Many jurisdictions monitored wastewater for SARS-CoV-2, the virus that causes COVID-19, and used this information to inform policy decisions.^1^ Some also publicly displayed that information to inform the public about community levels of infection.^2^

Ethical and legal expertise can usefully inform policy decisions about the public display of wastewater monitoring data. These decisions can sometimes involve conflicts between ethical values: for instance, public display of wastewater surveillance data may promote good governance and enable the public to better protect their health, but also raises privacy concerns and the possibility of stigmatization. In this Essay, we propose an ethics framework for considering whether and how to display information about pathogens detected in wastewater to the public. We then apply that framework to specific examples. We focus on public display of data, rather than the activity of wastewater monitoring itself, which has already prompted ethical analysis.^3^ The focus of these ethical analyses has generally been the collection of data from wastewater samples, rather than the display of already collected data.^4^ Although some authors have noted the communication of findings as ethically relevant, and others have proposed and implemented model data dashboards, public display has not been the focus of an independent ethical analysis.^5^

## I. ETHICAL VALUES

Four substantive values appear most relevant to decisions about public display of wastewater data. These values are relevant because of their centrality elsewhere in public health ethics and the connection between public health ethics writ large and the ethics of wastewater surveillance and of the public display of wastewater data in particular.^6^ The first is **benefiting people and preventing harm**. Publicly displaying wastewater monitoring data can benefit the public and empower them to make decisions that protect against health-related harm. For instance, residents could seek out testing based on wastewater pathogen data. It also, however, presents the risk of mistaken decisions taken in reliance on publicly displayed data. For example, if the public decides to avoid a neighborhood out of fear of contracting an infectious disease, even though the risk of infection is very low, this could deprive neighborhood residents of opportunities and subject them to stigma. Such an outcome would constitute harm due to the display of wastewater data information, understood as a setback to ethically important interests that would not likely have occurred without the data being displayed.

The second is **mitigating unfair disadvantage**. Pandemic diseases have imposed greater burdens on those who have faced unfair disadvantage such as racism.^7^ If public display of wastewater data helps reduce the burden of disease, this could also help mitigate unfair disadvantage.^8^ To effectively realize this goal, public display of data must be accessible and understandable to those who have faced unfair disadvantage. Policies must also consider potential ways in which the public display of wastewater data could exacerbate unfair disadvantages. For instance, if people avoid communities where infection levels appear higher, this could exacerbate disadvantage, for example by harming businesses in those areas. Net effect on disadvantage should be compared to the outcomes that would result if data were not publicly displayed. Mitigating disadvantage should not be equated with sameness of treatment. For instance, it could be appropriate to display data on certain pathogens and not others, or certain types of pathogen data and not others, if doing so achieves an optimal balance of benefit against potential harm or better serves the objective of mitigating disadvantage.

The third is **privacy**. While privacy can be valuable in view of its contribution to harm prevention and mitigating unfair disadvantage, many view privacy as additionally valuable in itself. In the context of public display of wastewater monitoring data, the clearest privacy challenges arise if specific individuals can be identified as carriers of pathogens.^9^ But problems could also arise for identification at the building or block level. These privacy issues could arise both in the form of private individuals making inferences about others’ infection status or behavior, and in the form of government officials or law enforcement making these inferences.^10^

The last is **autonomy**. As with privacy, autonomy can be valuable in view of its contribution to harm prevention and mitigating unfair disadvantage. But many view providing information that enables the public to decide for themselves as additionally valuable, even when other ways of protecting the public are available. Beyond individual autonomy, displaying information publicly can also foster collective autonomy or more informed civic decision making. It could allow the public to push for specific policies or critique decision makers who fail to adjust policies in response to data. Sometimes, different actors’ autonomy or different aspects of autonomy can conflict: for instance, some individuals’ autonomy might be advanced by data display even while others’ autonomy is set back.

In addition to these substantive values, there is also a relevant procedural ethical value, good governance.^11^ Like any governmental decision, decisions about public display of wastewater data should conform to elements of good governance: for example these decisions should be responsive to public values and informed by processes of community engagement, and the reasoning behind these decisions should be transparent. For example, in their study of the development and implementation of a wastewater dashboard system to track opioid use in Tempe, Arizona, Bowes et al. find that ‘community buy-in is key’ for developing an effective system.^12^ Good governance can also be promoted by public display of wastewater data: ideally, making wastewater data available to the public will in turn strengthen good governance by improving public knowledge of public health risks and the public’s ability to hold policymakers accountable. Additionally, public display of data could be a requirement of good governance because it could help provide the public with a justification for policies that government enacts—for instance, restrictions on travel. Good governance enables government to create an environment where the four substantive values can be effectively realized, allowing for individuals to flourish.

Ideally, good governance and public display of wastewater data can form a ‘virtuous cycle.’ In such a cycle, public display can help inform the public about public health risks, which in turns enables the public to make more informed and authoritative decisions about public health policies—including policies for the public display of data. Many frameworks emphasize that policies about wastewater surveillance should be developed in consultation with the affected public.^13^ Public display of data could help empower the public to more informedly engage in a consultation process.

## II. FACTORS TO CONSIDER WHEN MAKING PUBLIC DISPLAY DECISIONS

Some factors that may be relevant when deciding whether and how to publicly display wastewater data about the prevalence of a pathogen include (Table 1):

1) the total public health burden of the condition caused by the pathogen, including both individual severity and population-level harms such as exacerbated inequity
2) the extent to which a condition or geographic location is stigmatized
3) the likelihood that the public will reach correct conclusions based on data
4) whether the condition is easily communicable or poses a risk to others
5) the availability of discretionary actions members of the public can take in response

**Table 1:** An Ethics Framework for Public Display of Wastewater Data

**Factor**	**Underlying value(s)**	**Implications for display**
Public health burden of condition	Benefiting people and preventing harm; mitigating unfair disadvantage	Favors display if large
Stigma of condition or place	Mitigating unfair disadvantage; privacy	May disfavor display if display increases stigma or jeopardizes privacy
Public ability to understand data	Autonomy; Good governance	Favors display
Communicability of condition	Benefiting people and preventing harm	Favors display
Data’s usefulness in enabling protective action	Benefiting people and preventing harm	Favors display
Community support of data display	Good governance	Favors display

Any public display of wastewater data must be justified by the potential benefits, including public health benefits, the potential to mitigate unfair disadvantage, supporting individual autonomy, and promoting transparent and accountable decisionmaking. The potential public health benefits are likely to be more significant when the data concerns a pathogen that is easily communicable or poses a risk to others, and when there are actions that members of the public can take to protect themselves, such as vaccination. The more severe a condition is, the greater the potential benefits of publicizing wastewater data. Examples include respiratory viruses such as SARS-CoV-2 and influenza. These viruses can cause severe illness, are easily communicable, and their harmful impact can be mitigated by readily available steps the public can choose to take, such as vaccination and improved ventilation. Other pathogens that may fall into this category include insect-borne pathogens like West Nile virus, dengue, and malaria, and enteric pathogens like norovirus.

The potential harms of displaying data must also be considered, and these are likely to be greater when the health condition at issue is stigmatized or the public has a poor understanding of it. Examples in this category include sexually transmitted infections such as syphilis and human immunodeficiency virus (HIV). Along with potential stigma, public display of granular data about HIV or syphilis prevalence is less likely to advance the public’s ability to protect themselves, since these conditions are primarily communicable not through presence in a geographic location but rather through specific activities such as sexual intercourse or injecting drugs, and the risk of a specific sexual encounter or decision to inject drugs may not be closely tied to community-wide disease prevalence.

In considering stigma, public display decisions should realize that whether a pathogen or condition is stigmatized may shift over time and vary with context. For instance, SARS-CoV-2 is likely less stigmatized in the United States now that the vast majority of Americans have been infected. In contrast, mpox virus may be more stigmatized in the United States, where it is associated with stigmatized communities like gay men, than in communities abroad where it is considered primarily a zoonotic disease.^14^ Policymakers should also be aware of the possibility that public display of data may mitigate rather than worsen stigma: for instance, if it shows that mpox virus prevalence is low across all geographic communities (including those with a higher percentage of gay men), or that SARS-CoV-2 prevalence is not associated with any specific community or group.

How to consider stigma may also depend on the localized persistence of risk. Although many pathogens, such as COVID-19 or influenza, may have transient spikes, insect-borne pathogens or environmental toxins may be longer lasting. The persistent presence of a health threat could lead to a location, rather than a population, becoming stigmatized.^15^ Stigmatization, however, should not automatically be attributed to the public display of wastewater data on a pathogen or toxin, nor should concerns about stigma warrant concealment. The public is likely to become aware of a hazard in other ways, making it more appropriate to provide the public with accurate information while working to support individuals and communities whose opportunities are being narrowed by stigma.

Complex issues arise when a pathogen is severe but also misunderstood. Examples may include Ebola virus disease or plague. While these conditions are severe and communicable, there are fewer targeted actions the public can take in response to data on cases. Any action taken may only be marginally effective, meaning that the public benefit will be small and could be outweighed by harms such as panic. In some cases, public display of data could produce ‘run on the bank’ phenomena that are individually rational but collectively irrational, for instance if people attempt to flee a geographic area in response to publicly displayed survey data showing a spike in a dreaded communicable condition, but experience greater harm because fleeing exposes them to injury and disrupts needed medical care for existing conditions and support for other needs such as child care. Other conditions, such as pathogens that selectively impact certain population groups like children or older adults, may prompt a heterogeneous response, with some members of the public changing their behavior substantially and others not doing so at all. These possibilities warrant careful social-scientific study and analysis, which should inform decisions about how best to display data to the public.

While our interest is primarily in public display of pathogen data in wastewater, other types of data can also be collected from wastewater. Examples include data on levels of opioid metabolites.^16^ These health risks are not communicable, reducing the urgency of displaying this data publicly. There may be other reasons to display information about noncommunicable health risks, however, including informing the public about health inequities. Our framework is applicable to the public display of data on these other risks as well, and factors other than communicability could justify publicly displaying this data—for instance, if wastewater data allows individuals to avoid consuming contaminated drinking water.

This framework offers policymakers a comprehensive overview of the factors they should consider when deciding whether to publicly display wastewater data and when these factors speak in favor or against public display. Our hope is that it provides policymakers with a tool for considering multiple values and considerations, identifying potential conflicts between them, considering how to weigh them in cases of conflict, and arriving at a defensible decision.

## III. IMPLEMENTATION CONSIDERATIONS

In publicly displaying wastewater data, it will be important to use a display modality that is easily accessible to all, including: people who only access data via mobile devices, people who do not read English, people with visual and other disabilities, and people who have limited familiarity with data.^17^ It is also important to consider how specific communities may be excluded from the benefits of publicly displayed wastewater data: for instance, rural or low-population areas may not use sewer systems, and some poorer or unhoused people even in populous areas may not use toilets connected to sewersheds.^18^

Values also have implications for how data is displayed, not merely whether it is displayed. More granular data may be more useful at informing a wider range of decisions, but could present greater risks of identification. Some of the same issues around privacy protections and data display that have arisen in discussions around the display of Census data may also be relevant to wastewater data (Kenny et al. 2021).^19^

It will also be important to consider whether data should be presented in similar formats for everyone, or whether certain data should be limited or targeted to experts using it for research purposes versus the general public. Data dashboard implementation proposals have suggested, for instance, excluding building-level maps from publicly available dashboards and instead aggregating only at a higher level (such as a neighborhood), while allowing public health departments to view more granular data.^20^ Data display should be designed to reduce the risk of data misinterpretation.

Though the framework’s fundamental objectives should remain constant irrespective of where data is being collected, details will affect implementation. For instance, as noted above, building-level data collection heightens concerns about privacy and could disfavor broad public display of building-level data.

The framework we have presented focuses on ethics, which can help to guide governance and the creation of legal obligations, but is not equivalent. For instance, while the usefulness of data in promoting protective action ethically favors its display, we do not propose to mandate the display of useful data or to legally prohibit the display of data that is unlikely to be useful. As such, our framework does not propose that a specific body be responsible for data collection and oversight, or propose specific arrangements for private-sector data display or for its prohibition. However, to the extent that the fundamental ethical objectives we identify here have already been translated into legal rights or obligations in certain jurisdictions, those legal rights and obligations may be particularly relevant to decisions around the display of wastewater surveillance data. For instance, freedom of information provisions premised on the importance of autonomy might undergird legal duties to make information publicly accessible, whereas privacy rights might constrain the display of other information. Though privacy rights provide relevant constraints, they are unlikely to justify allowing an individual or group to block the display of deidentified data.

## IV. CONCLUSION

The COVID-19 pandemic has underscored the significance of wastewater monitoring in identifying the presence of pathogens and guiding policy decisions. The public display of this information has proven to be an effective tool in informing communities about community levels of infectious disease.^21^ However, the ethical and legal implications surrounding the public disclosure of wastewater monitoring data cannot be overlooked. To address this, our proposed ethics framework provides a valuable resource for policymakers to navigate the complexities associated with displaying such information. By considering the framework's guidelines, policymakers can strike a balance between multiple values. Implementing this framework will bring greater rigor and clarity to decisions about the public display of wastewater surveillance data.

## ACKNOWLEDGMENTS

Thanks to Natalie Ram and Nathan LaCross for helpful discussion during early stages of this project, and to Liz Ignowski and Halli Berebbi for research assistance.

## Footnotes

1 Meri R. J. Varkila et al., *Use of Wastewater Metrics to Track COVID-19 in the US*, 6 JAMA Netw. Open e2325591 (2023).

2 Erin G. Wettstone et al., *Interactive SARS-CoV-2 Dashboard for Real-Time Geospatial Visualisation of Sewage and Clinical Surveillance Data from Dhaka, Bangladesh: A Tool for Public Health Situational Awareness*, 8 BMJ Glob. Health (2023), https://gh.bmj.com/content/8/8/e012921 (last visited Mar. 19, 2025).

3 Natalie Ram et al., *Ethical and Legal Wastewater Surveillance*, 379 Science 652 (2023); Steve E. Hrudey et al., *Ethics Guidance for Environmental Scientists Engaged in Surveillance of Wastewater for SARS-CoV-2*, 55 Environ. Sci. Technol. 8484 (2021).

4
*E.g.* Teresa Scassa, Pamela Robinson & Ryan Mosoff, *The Datafication of Wastewater: Legal, Ethical and Civic Considerations*, 2022 Technol. Regul. 23, 23–35 (2022); Janelle R. Thompson et al., *Making Waves: Wastewater Surveillance of SARS-CoV-2 for Population-Based Health Management*, 184 Water  Res. 116181 (2020).

5 Mhara M. Coffman et al., *Preventing Scientific and Ethical Misuse of Wastewater Surveillance Data*, 55 Environ. Sci. Technol. 11473, 11473–75 (2021); Hrudey et al., *supra* note 3; Dustin Hill et al., *Operationalizing an Open-Source Dashboard for Communicating Results of Wastewater-Based Surveillance*, 11 MethodsX 102299 (2023).

6 Ezekiel J. Emanuel & Govind Persad, *The Shared Ethical Framework to Allocate Scarce Medical Resources: A Lesson from COVID-19*, 401 The  Lancet 1892, 1892–902 (2023); Nancy McClung et al., *The Advisory Committee on Immunization Practices’ Ethical Principles for Allocating Initial Supplies of COVID-19 Vaccine-United States, 2020*, 21 Morbidity and  Mortality weekly report 1782, 1782–86 (2020).

7 Lakshmi Krishnan, S. Michelle Ogunwole & Lisa A. Cooper, *Historical Insights on Coronavirus Disease 2019 (COVID-19), the 1918 Influenza Pandemic, and Racial Disparities: Illuminating a Path Forward*, Ann. Intern. Med. 474, 474–81 (2020).

8 Clara Y. Medina et al., *The Need of an Environmental Justice Approach for Wastewater Based Epidemiology for Rural and Disadvantaged Communities: A Review in California*, 27 Curr. Opin. Environ. Sci. Health 100348 (2022).

9 Lance Gable, Natalie Ram & Jeffrey L Ram, *Legal and Ethical Implications of Wastewater Monitoring of SARS-CoV-2 for COVID-19 Surveillance*, 7 J. Law  Biosci. lsaa039 (2020).

10 Dhiraj Nainani et al., *Balancing Public Health and Group Privacy: Ethics, Rights, and Obligations for Wastewater Surveillance Systems*, 258 Water  Res. 121756 (2024).

11 Ole F. Norheim et al., *Difficult Trade-Offs in Response to COVID-19: The Case for Open and Inclusive Decision Making*, 27 Nat. Med. 10, 10–13 (2021); Michelle M. Mello & C. Jason Wang, *Ethics and Governance for Digital Disease Surveillance*, 368 Science 951, 951–4 (2020).

12 Devin A. Bowes et al., *Leveraging an Established Neighbourhood-Level, Open Access Wastewater Monitoring Network to Address Public Health Priorities: A Population-Based Study*, 4 Lancet  Microbe e29 (2023); Bethany Cooper et al., *Maintaining a Social License to Operate for Wastewater-Based Monitoring: The Case of Managing Infectious Disease and the COVID-19 Pandemic*, 320 J. Environ. Manage. 115819 (2022).

13
*E.g.* Hrudey et al., *supra* note 3.

14 Oriol Mitjà et al., *Monkeypox*, 401 The  Lancet 60, 60–74 (2023).

15 Nainani et al., *supra* note 10.

16 Bowes et al., *supra* note 12.

17 Jimmy Phuong et al., *Telehealth and Digital Health Innovations: A Mixed Landscape of Access*, 2 PLOS Digit. Health e0000401 (2023).

18 Alasdair Cohen et al., *Making Waves: The Benefits and Challenges of Responsibly Implementing Wastewater-Based Surveillance for Rural Communities*, 250 Water  Res. 121095 (2024).

19 Christopher T. Kenny et al., *The Use of Differential Privacy for Census Data and Its Impact on Redistricting: The Case of the 2020 U.S. Census*, 7 Sci. Adv. eabk3283 (2021).

20 Hill et al., *supra* note 5.

21 Emily Harris, *Wastewater Surveillance Predicts Large-Scale Flu, RSV Trends*, 330 JAMA 1422 (2023).

